# The new Veterinary Medicines Regulation: rising to the challenge

**DOI:** 10.1186/s13620-022-00209-6

**Published:** 2022-02-03

**Authors:** Simon J. More, Finola McCoy, Catherine I. McAloon

**Affiliations:** 1 Centre for Veterinary Epidemiology and Risk Analysis, UCD School of Veterinary Medicine, University College Dublin, Belfield, Dublin, D04 W6F6 Ireland; 2 UCD School of Veterinary Medicine, University College Dublin, Belfield, Dublin, D04 W6F6 Ireland; 3 Animal Health Ireland, 4-5 The Archways, Carrick on Shannon, Co., Leitrim, N41 WN27 Ireland

**Keywords:** Veterinary Medicines Regulation, Regulation (EU) 2019/6, Intramammary antimicrobials, Antimicrobial stewardship, Mastitis control, Private veterinary practitioners, Ireland

## Abstract

This article focuses on the new Veterinary Medicines Regulation, which is applicable across all Member States of the European Union, including Ireland, from 28 January 2022. From this date, prophylactic use of antimicrobials (AMs) in groups of animals is banned, metaphylactic use in groups of animals is restricted, and certain AMs are reserved for humans only. In the Irish dairy industry, as elsewhere, successful implementation of the Regulation will require a high level of mastitis control across all herds, and measures to support high standards in antibiotic stewardship. National actions will be critical, to support optimal mastitis control throughout the national herd. For private veterinary practitioners (PVPs), the Regulation will lead to specific prescribing changes, including the requirement to shift from blanket to selective dry cow therapy. Further, prescribing choices will need to be guided by the categorisation for AMs developed by the European Medicines Agency (EMA). More broadly, the Regulation requires a fundamental shift in thinking both in terms of AM usage and of the role of the PVP. Given the close association between mastitis control and intramammary AM stewardship, it is imperative that prescribing and mastitis control decisions are made concurrently. A herd health approach will be critical, within a Client-Patient-Practice Relationship as outlined by the Veterinary Council of Ireland. On those farms with sub-optimal mastitis control, mastitis issues need to be sustainably resolved. A detailed farm investigation by the PVP, in partnership with the farmer and other milk quality professionals, is essential, to understand the epidemiology and on-farm drivers of mastitis, to develop farm-specific action plans, and to facilitate ongoing monitoring of progress. It is vital that PVPs provide leadership, with the provision of a holistic, herd health approach to inform both prescribing and mastitis control decisions in herds under their care.

This article focuses on the new Veterinary Medicines Regulation (Regulation 2019/6,[1]), including national measures to support on-farm antimicrobial (AM) stewardship, and challenges and opportunities for Irish private veterinary practitioners (PVPs). We particularly focus on the prescribing of intramammary AMs in dairy practice, but the information is relevant more broadly. This Commentary provides a brief overview of the key issues, with more detail provided elsewhere (More SJ, Buckley W, Downing K, Kelly P, McAloon C, McGrath M, O’Grady L, O’Sullivan F, Ryan EG, Silva Boloña P, McCoy F: Intramammary antibiotic stewardship in the Irish dairy industry: challenges and opportunities, under review).

## The Veterinary Medicines Regulation

The Veterinary Medicines Regulation was adopted by the European Parliament and the European Council in December 2018 and is applicable in all Member States, including Ireland, from 28 January 2022. There are key changes for PVPs working in dairy practice throughout the European Union. In particular, prophylactic use 1 of AMs in groups of animals is banned, metaphylactic use 2 in groups of animals is restricted, and certain AMs are reserved for humans only. At a broader level, member states are required to collect data on the sale and use of AMs in animals.

These changes are motivated by the urgent need to sustainably address AM resistance (AMR), which is recognised as a key global challenge affecting human, animal, plant and environmental health. It is well recognised that this challenge can only be tackled with a holistic and multisectoral One Health approach across all relevant sectors, including animal health. As part of the Farm to Fork Strategy within the EU Green Deal, there is a commitment to action to reduce overall sales of AMs for farmed animals and in aquaculture by 50% by 2030 [2]. These policy actions may seem somewhat remote for the ordinary citizen, but fundamentally they are seeking to ensure availability of effective AMs for future generations.

At this point, it is useful to clarify the usage of ‘therapeutic’, ‘metaphylactic’ and ‘prophylactic’ in the context of intramammary AMs. During lactation, intramammary therapy is primarily therapeutic. Metaphylactic use may be indicated in response to a large outbreak of highly contagious mastitis, but this is a very rare event, and generally associated with suboptimal hygiene, milking routine or farm management. At drying off, therapy can be either therapeutic (if a cow is infected at the point of drying off) or prophylactic (if the cow is not infected at this point). Dry cow therapy (DCT) cannot be considered metaphylactic because the milking process (the primary risk factor for spread of contagious mastitis) has been removed at the point of drying off.

## National measures to support on-farm AM stewardship

There are two factors that are critical to successful implementation of the Veterinary Medicines Regulation within the Irish dairy industry, including a high level of mastitis control across all herds, and measures to support high standards in antibiotic stewardship. Both of these factors are needed, as good farm-level mastitis control is a prerequisite for key strategies that underpin intramammary AM stewardship, including a reduced reliance on AMs and a reduction in the risk associated with the shift from blanket to selective DCT.

We currently estimate that about two-thirds of Irish herds have optimal mastitis control (that is, 65% of Irish herds in 2020 had an annual unadjusted geometric mean somatic cell count (SCC) of 200,000 cells/mL or less (More SJ, Buckley W, Downing K, Kelly P, McAloon C, McGrath M, O’Grady L, O’Sullivan F, Ryan EG, Silva Boloña P, McCoy F: Intramammary antibiotic stewardship in the Irish dairy industry: challenges and opportunities, under review). AM stewardship is much more challenging in the remaining one-third of Irish herds with suboptimal mastitis control. In these herds, there are more infected cows, and often lower levels of hygiene and farm management and increased infection challenge at all stages of production. In these situations, it can be more difficult to protect non-infected cows. In these herds, there is currently a reliance on DCT to resolve infection, both to treat cows infected at the end of lactation (therapeutic usage) and to prevent new infection during the dry period (prophylactic usage).

In the years prior to 2017, considerable progress was made to improve national milk quality, particularly through the national CellCheck programme, coordinated by Animal Health Ireland. However, progress has now stalled, and there has been no substantive improvement in national milk quality since 2017. National leadership is needed, both from industry and government, to leverage additional ‘drivers’ for improved milk quality. In particular, opportunities exist with respect to regulation, the Bord Bia Sustainable Dairy Assurance Scheme (SDAS), and national research. National and EU legislation is currently being interpreted in a manner that facilitates ongoing supply, with minimal requirement to sustainably resolve mastitis issues. This is explained in detail elsewhere (More SJ, Buckley W, Downing K, Kelly P, McAloon C, McGrath M, O’Grady L, O’Sullivan F, Ryan EG, Silva Boloña P, McCoy F: Intramammary antibiotic stewardship in the Irish dairy industry: challenges and opportunities, under review), but as one example, during the first 6 months following a one-month break-in-supply, all Irish farms are eligible to supply raw milk for processing of dairy products regardless of the bulk tank SCC [3]. Similarly, although SDAS has the potential to motivate farmers towards improved milk quality, this potential has not yet been realised, as the SDAS standards do not exceed the legislative baseline with respect to milk quality [4]. Finally, there is a need for a detailed understanding of opportunities and constraints to improved mastitis control in the national herd. Currently, however, national research is constrained as access to key bulk tank SCC data is limited.

The stewardship of on-farm intramammary AMs is challenging, particularly given the history of remote prescribing in Ireland. Since 2007, national legislation has been in place to allow veterinarians to prescribe intramammary AMs without a requirement for a herd visit at least every 12 months [5, 6], provided the herdowner is participating in a mastitis control programme coordinated by the milk processor. This programme is required to be in writing, and includes an outline of the specific requirements relating to mastitis control that the milk processor, the farmer and the veterinarian must each meet. This remote (or so-called Schedule 8) prescribing is estimated to account for approximately 30% of all tubes prescribed for dry-cow therapy. There are ongoing concerns about the application of remote prescribing in Ireland, including by McAloon et al. ([7]) who suggested that ‘this prescribing route is unlikely to provide the veterinary oversight necessary to support prudent prescription decision making on the basis of a detailed, on-farm understanding of mastitis and farm management’. AM stewardship is further compromised by the potential for Irish farmers to source intramammary AMs from multiple sources.

To this point, Ireland has lagged behind international competitors with respect to national measures to support on-farm AM stewardship. However, this is rapidly changing, in part due to the introduction of the Veterinary Medicines Regulation, and facilitated by the establishment of *i*NAP, Ireland’s National Action Plan on Antimicrobial Resistance, spanning human and animal health and the environment [8]. Key changes include a revision by the Veterinary Council of Ireland of their Code of Professional Conduct and the establishment of a national e-prescribing database by the Department of Agriculture, Food and the Marine. In a number of European countries, these databases are being used to benchmark AM prescribing and usage, nationally, by sector, and also by individual PVPs and farmers, with the potential to positively influence standards in AM stewardship [9]. International experience will also be useful to inform further national measures, including the potential for sector-wide bans (either voluntary or mandatory) on the use in animals of AMs of highest priority for public health, as well as the development of treatment guidelines to support clinical decision-making (More SJ, Buckley W, Downing K, Kelly P, McAloon C, McGrath M, O’Grady L, O’Sullivan F, Ryan EG, Silva Boloña P, McCoy F: Intramammary antibiotic stewardship in the Irish dairy industry: challenges and opportunities, under review).

## Challenges and opportunities for Irish PVPs

The Veterinary Medicines Regulation will lead to specific prescribing changes, most notably the requirement to shift from blanket to selective DCT. In other words, DCT must be limited to those animals that are infected at the point of drying off. Concurrently, on-farm hygiene standards will become paramount with increased reliance on sealant-only products. Further, the choice of prescribing AM will need to be guided by the categorisation for AMs developed by the European Medicines Agency (EMA), in terms of AM resistance risk to human health, either category A (*‘Avoid’*), B (*‘Restrict’*), C (*‘Caution’*) or D (*‘Prudence’*). By definition, Category A AMs should be avoided in dairy practice. Category B (including 3rd and 4th generation cephalosporins) should not be used as first line treatments, and only used for treatment when there is no alternative AM in a lower category (C or D) shown to be clinically effective based on the results of AM sensitivity testing [10]. The widespread, and increasing, use of 3rd and 4th generation cephalosporins (Category B AMs) in Ireland, both for in-lactation therapy and DCT [7] is of particular concern, and at odds with these guidelines. Analyses of the national intramammary AM sales data from 2020 have confirmed these trends (McAloon CI, McCoy F, More SJ: Intramammary antimicrobial sales in Ireland: a 2020 update, under review). In addition, under the new Regulation, AMs should only be prescribed for the duration of treatment, with a prescription remaining valid for 5 days. This will require a change in how lactating cow AMs are prescribed and managed, with the development and review of annual herd-specific treatment plans offering a practical solution to some of the logistical challenges of the new Regulation.

More broadly, the Regulation is requiring a fundamental shift in thinking both in terms of AM usage and of the role of the PVP. The legislation explicitly states that AMs *‘shall not be applied routinely nor used to compensate for poor hygiene, inadequate animal husbandry or lack of care or to compensate for poor farm management’.* Similarly, there is a need to recognise the close association between mastitis control and intramammary AM stewardship, and for prescribing and mastitis control decisions to be made concurrently. As well as being essential, it is an opportunity for PVPs to engage with and become more involved with their clients’ mastitis management. A herd health approach is critical, within a Client-Patient-Practice Relationship (CPPR) as outlined by the Veterinary Council of Ireland's Code of Professional Conduct for Veterinary Practitioners [11]. It also requires the PVP to have a sophisticated understanding of the farm, including the herd, the people, the facilities, and aspects of farm management relevant to mastitis control. Individual animal information, herd-level information and EMA guidelines will each contribute to prescribing decisions. Individual animal information is needed, preferably milk recording, for PVPs to both prescribe responsibly but also to understand and monitor udder health in their clients’ herds. All information sources are diagnostically imperfect, and PVPs will need to utilise a risk-based approach to evaluate the infection status of individual cows at drying off. With respect to herd-level information, PVPs need a thorough farm-specific understanding of milk quality trends, the mastitis pathogen challenge, and AM sensitivity/resistance patterns. To enable this, PVPs need to be familiar with and skilled in their interpretation of milk recording data. The CellCheck dashboard, available through the ICBF website, is a critical resource, and allows the user to interact with milk recording data to identify and monitor SCC patterns over time, and in animal groups. Other resources, decision-support tools and training are available, or under development, from the CellCheck technical working group [12]. The EMA guidelines are freely available [10].

On those farms with sub-optimal mastitis control, mastitis issues need to be sustainably resolved. A detailed farm investigation by the PVP is essential, in partnership with the farmer and other milk quality professionals, consistent with the CellCheck multi-disciplinary model, to understand the epidemiology and on-farm drivers of mastitis, to develop farm-specific action plans, and to facilitate ongoing monitoring of progress. Herds with chronic, seemingly intractable, mastitis problems are a particular challenge. As highlighted previously, sustainable solutions on these farms may not be possible without changes to the interpretation of national and EU legislation, to ensure there is a regulatory imperative for herds to fundamentally address underlying factors that contribute to suboptimal mastitis control.

## Conclusion

The new Veterinary Medicines Regulation reflects scientific and societal concerns of the risk posed to public health by AM resistance, and provides a framework for best-practice in AM stewardship in food animal production. In Ireland, there are both challenges and opportunities in the coming period with the introduction of this Regulation. A substantial shift from current practice will be needed, both for PVPs and farmers, noting that there have been only tentative steps to this point towards selective DCT, a worrying recent upward drift in national sales of EMA Category B intramammary AMs (3rd and 4th generation cephalosporins) [7], and ongoing limited penetration of milk recording across the national herd [13]. Education will be critical to ensure a smooth transition. It is vital that PVPs provide leadership, with the provision of a holistic, herd health approach to inform both prescribing and mastitis control decisions in the herds under their care. PVPs play a central role, as AM stewards, in the Irish dairy industry into the future.

## Data Availability

Not relevant.

## Footnotes

1 In EU Regulation 2019/6, prophylactic use is defined as *‘the administration of a medicinal product to an animal or group of animals before clinical signs of a disease, in order to prevent the occurrence of disease or infection’.*

2 In EU Regulation 2019/6, metaphylactic use is defined as *‘the administration of a medicinal product to a group of animals after a diagnosis of clinical disease in part of the group has been established, with the aim of treating the clinically sick animals and controlling the spread of the disease to animals in close contact and at risk and which may already be subclinically infected’.*
